# Increased cancer incidence among patients with hidradenitis suppurativa – a Danish nationwide register study 1977–2017

**DOI:** 10.2340/1651-226X.2024.26182

**Published:** 2024-04-21

**Authors:** Rune Kjærsgaard Andersen, Klaus Rostgaard, Ole Pedersen, Gregor Borut Ernst Jemec, Henrik Hjalgrim

**Affiliations:** a Department of Dermatology, Zealand University Hospital, Roskilde, Denmark;; b Department of Immunology and Microbiology, Skin Immunology Research Center, University of Copenhagen, Denmark;; c Danish Cancer Institute, Danish Cancer Society, Copenhagen, Denmark;; d Department of Epidemiology Research, Statens Serum Institut, Copenhagen, Denmark;; e Department of Clinical Immunology, Zealand University Hospital, Køge, Denmark;; f Department of Clinical Medicine, Faculty of Health and Medical Science, University of Copenhagen, Denmark; g Department of Haematology, Rigshospitalet, Copenhagen, Denmark

**Keywords:** Hidradenitis suppurativa, cancer, cancer risk, registries, epidemiology

## Abstract

**Background:**

The chronic, inflammatory skin disease hidradenitis suppurativa (HS) (prevalence: 0.5%–1%, diagnostic delay: 7–10 years) primarily arises in younger adults and frequently coincides with autoimmune comorbidities and unhealthy life-styles (smoking and obesity). These factors are known to increase cancer risk, but despite this, information on cancer occurrence among HS patients is scarce.

**Materials and methods:**

A nationwide retrospective register-based study assessing relative risk of cancer – overall and by anatomical site – following HS diagnosis expressed as standardized incidence ratios (SIRs), which is ratios between observed cases among all Danes diagnosed with HS since 1977 and expected cases based on cancer incidence rates of the entire Danish population during the same period.

**Results:**

Participants consisted of a cohort of 13,919 Danes with HS, who during an average of 14.2 years of follow-up developed a total of 1,193 incident cancers, corresponding to a 40% increased risk (SIR = 1.4, 95% CI: 1.3 to 1.4, *p* < 0.001). Increased risks were observed for cancers of the respiratory system, oral cavity and pharynx, digestive organs and peritoneum, urinary tract, and the lymphatic tissues.

**Interpretation:**

These findings underline an unmet need for health monitoring, lifestyle interventions and cancer screening if and when relevant.

## Background

**Figure 1:**
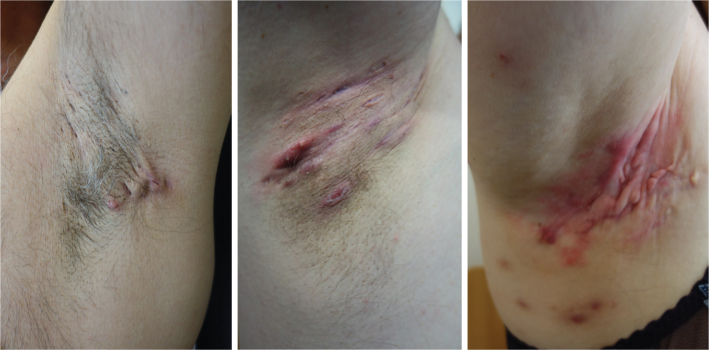
Examples of axillary hidradenitis suppurativa’s clinical presentation. Thee illustrations of increasingly severe axillary hidradenitis suppurativa. All cases show characteristic morphological signs consisting of relevant lesions and various degrees of cutaneous inflammation and scarring.

Hidradenitis suppurativa (HS) is a severe, chronic, inflammatory skin disease, presenting with painful nodules and abscesses of a non-infectious etiology that primarily affect young women (female/male ratio 3:1). The lesions may evolve into suppurating skin tunnels (Figure 1), where subsequent immunomodulation can result in considerable restrictive scaring of affected areas [1]. Treatment options are limited, but includes the use of anti-inflammatory agents, and/or surgical removal of the lesion(s) [1, 2]. HS etiology remains elusive, but underlying immune dysfunction is suspected due to the high prevalence of a wide variety of metabolic and autoimmune comorbidities among HS patients [3–6].

Prevalence estimates for HS vary from 0.1%–0.5% to 1%–2% [7–9] based on the origin/case definition used (hospital-based register data vs. self-reported information on symptoms). Patients with HS often have low socioeconomic status [10, 11] and are known to be at a higher risk of tobacco smoking, alcohol consumption and obesity [3, 4, 12, 13]. These factors constitute some of the most important environmental risk factors for cancer, and with a typical onset of HS around the second to third decade of life [1] – a time-period when people are more focused on finding their way through life and less concerned about long-term consequences of life style choices – a patient group at particular risk of future cancer development is formed.

Despite such manifest risk exposures, the scope of prior cancer research within the HS field has been narrow, for example: the development of cutaneous squamous cell carcinoma within HS lesions [14]. In fact, only two register studies, one Swedish [15] and one Korean [16], have investigated HS-related cancer risk at a population level. While both studies indicated an increased risk of cancer among patients diagnosed with HS, they disagreed widely upon the risk-types of cancer. Furthermore, these studies were hampered by either small number of outcomes, that is cases of cancer (*N* = 73 [15]) or methodological/cohort-based shortcomings (undisclosed follow-up time, male-dominant study cohort un-representative of traditional HS populations [16]). We therefore took advantage of nation-wide data on hospital discharge and cancer diagnoses to assess overall as well as site-specific cancer risks among residents in Denmark diagnosed with HS in the period 1977 to 2017.

## Materials and methods

### Information on Danish National Health Registries

Data for this nation-wide register-based study were collected by taking advantage of the nation-wide Danish Civil Registration System (CRS) implemented on April 1, 1968, when all Danish citizens were assigned unique identification numbers (the CRS number). Since then, all individuals living in Denmark for at least three consecutive months have been assigned a CRS number, which also function as the obligatory means of identification for all important contacts with the private/public sector, for example, property purchases, housing registration, job hiring, tax forms, all medical treatment (both for identification and billing purposes) etc. As such, for all practical purposes, the coverage of the CRS is considered complete. The CRS number also allows for identity-secure linkage between health registers [17], where it is used to track migration, death and health status as provided by, for example, the Danish Cancer Register (CAR) [18] and the Danish National Patient Register (DNPR) [19].

In Denmark, data on all hospital-based medical diagnoses are registered in DNPR, implemented nation-wide in January 1977 [19]. Information is submitted from all hospitals on a weekly basis, and link patient CRS number to the day of admission/discharge/outpatient contact, department, operations and the International Code of Diseases (ICD) code giving rise to the hospital contact. In January 1994 DNPR switched to registering diseases based on ICD-10 instead of ICD-8, and at the same time, data from outpatient records and emergency departments were also included in the DNPR [20]. For this study, we identified all individuals with a diagnosis of HS (ICD8: 705.91, ICD10: L73.2) between 1977 and 2017, and defined their diagnosis time of HS as the first date of a hospital contact including an ICD code for HS.

Cases of cancer diagnosed among patients diagnosed with HS were identified through linkage with CAR using the CRS number as key. CAR includes information on all incident cancer cases in Denmark since 1943 with registered data including date of diagnosis and cancer type among others; see [18] and the documentation on the web on esundhed.dk for additional information. From this data, we created background cancer incidence rates for 71 non-overlapping types of cancer defined for statistical purposes by the Danish Health Authorities. All cancers were morphologically defined by WHO standards and mapped to an ICD code by the reporting health professional responsible for reporting the instance of cancer to CAR. As the only deviation from the implied categorization and aggregation in this standard, we grouped D459 (polycythemia vera), D471 (chronic myeloproliferative disease), D473 (essential [hemorrhagic] thrombocythemia), and D475 (hypereosinophilic syndrome) with the main group of blood forming tissue rather than the main group of lymphatic cancers. Likewise, 16 aggregated cancer forms and a combined group of ‘any cancer’ were defined from the 71 subgroups. National Danish background population cancer incidence rates were calculated for the same period by dividing the number of incident cancer ‘X’ cases from CAR in strata defined by sex, age group (0–4, 5–9, …, 95–99, 100+ years) and calendar period (1978–1982, 1983–1987, …, 2013–2017) with correspondingly stratified time at risk of cancer ‘X’ among all persons in Denmark, that is among persons alive and without cancer ‘X’ according to the registers.

### Statistical methods

We followed all persons with HS while alive and resident in Denmark from time of HS diagnosis or 01 January 1977 whichever occurred later to cancer diagnosis or the end of 2017 whichever came first for each of the cancer forms defined above.

Expected counts of cancer cases for the HS cohort were obtained as sums of age- period- and sex-specific total follow-up time (person-years at risk) multiplied by the correspondingly stratified cancer incidence rates in the background population based on CAR strata.

Standardized incidence ratios (SIRs), that is ratios between the observed and expected counts of incident cancers were analyzed using Poisson regression [21]. Analyses were carried out for cancer overall and for all individual non-overlapping cancer types in which ≥5 instances of cancer were reported during follow-up. Confidence intervals and *p*-value assessments were likelihood-ratio based.

Statistical significance was reported in the main table in a three-tier system: two-tailed *p* < 0.05 (*), two-tailed *p* < 0.05 corrected for multiple testing (CFMT) using the Bonferroni method (**) and *p* < 10^-6^ (***).

All datasets were created and statistical analyses performed using SAS Statistical Software (SAS Institute, Cary, NC. Version 9.4) and the stratify macro [21].

### Potential sources of bias

Individual level information on smoking, alcohol consumption, obesity, and HS severity, found traditionally in medical charts, are not reported to the Danish registries, and thus not accessible for analyses. An intuitive measure of HS disease severity can however be assessed by summating the number of DNPR diagnosis codes of HS for each citizen, as that provides the number of times each citizen has had a hospital contact based on HS.

To limit the effect of bias caused by HS severity we conducted a sensitivity analysis by dichotomized number of HS visit and provide hazard ratios (HRs) for those with ≥5 HS-related visits as compared to those with <5 visits. We did this for cancer overall and for those main groups of individual cancer types that had been found to reach statistical significance in the previous analysis, further restricted by the requirement for ≥5 observed cancers in both exposure groups. Using more elaborate measures of HS severity, taking into account the intensity of HS-related visits over shorter period of time, for example, a year were tested, but ultimately, for statistical reasons, did not lead to new insights and therefore, are not presented.

## Results

### Participants

Between 1977 and 2017 we followed 13,919 HS patients for diagnosis of incident cancers through an average of 14.2 years, 69.8% (9,709/13,919) of whom were female. Median age at HS diagnosis was 35 years (interquartile range 26 to 44 years), a little lower in females (Table 1).

**Table 1:** Characteristics of follow-up time in Danish citizens with HS.

HS age [years]	Males				Females			
	*N*	%	Total pyrs	Mean pyrs	*N*	%	Total pyrs	Mean pyrs
00–19	218	5.2	3,503	16.1	733	7.6	11,869	16.2
20–29	1,062	25.2	16,688	15.7	2,789	28.7	46,780	16.8
30–39	1,133	26.9	17,055	15.1	2,868	29.5	44,463	15.5
40–49	918	21.8	11,424	12.4	2,037	21.0	26,365	12.9
50–59	524	12.5	4,996	9.5	894	9.2	9,880	11.1
60–69	269	6.4	1,826	6.8	270	2.8	2,427	9.0
70+	86	2.0	444	5.2	118	1.2	616	5.2
total	4,210	100.0	55,936	13.3	9,709	100.0	142,400	14.7

Follow-up time times by sex and age at HS diagnosis (HS age). Median, lower and upper quartile of HS age was (36, 28, 47 years) in males, (34 s, 26, 43 years) in females and (35, 26, 44 years) overall. Overall *N* = 13,919, total person-years = 198,336 and mean person-years = 14.2. The mean year of entry into the study is the beginning of 2002.

N: number; HS: hidradenitis suppurativa; pyrs: person-years.

### Temporal variation in SIR by time since HS diagnosis and sex

Table 2 illustrates the common epidemiological phenomenon of an excess risk of cancer diagnosis following contact with the health system. Specifically, SIR of cancer was 2.8 in the half year following HS diagnosis falling to half that during longer follow-up. This early increase in cancer risk is typically attributed to surveillance bias or reverse causality and is difficult to interpret in a meaningful way. We therefore disregarded the first year of follow-up after HS diagnosis in the rest of our analyses. We found a mild to moderate variation in SIR based on sex, with estimates after the first year of HS diagnosis being 1.5 (95% CI: 1.4; 1.7) in males and 1.3 (95% CI: 1.2; 1.4) in females (*p* = 0.0022). Due to small numbers of observed cancers and absence of predefined sex-specific hypotheses, we do not present sex-specific numbers in the following.

**Table 2:** Observed and expected cancers of any type in Danish citizens with HS by time since HS diagnosis.

Time since HS	obs	exp	SIR
0–5 months	49	17.64	2.8 (2.1–3.6)
6–11 months	25	17.68	1.4 (0.9–2.0)
12–23 months	44	35.55	1.2 (0.9–1.6)
2–4 years	167	105.94	1.6 (1.3–1.8)
5–9 years	235	174.40	1.3 (1.2–1.5)
10+ years	747	565.57	1.3 (1.2–1.4)
All	1,267	916.78	1.4 (1.3–1.5)

SIR is calculated as obs/exp with 95% confidence limits.

HS: hidradenitis suppurativa; exp: expected number of incident cancers; obs: observed number of incident cancers; SIR: standardized incidence ratio.

### Cancer risk in patients diagnosed with HS

The analysis showed that patients with HS had an overall increase in cancer risk of 40% (SIR = 1.4, 95% CI: 1.3; 1.4, *p* < 1 × 10^-6^). This reflected statistically significantly increased risks of malignancies across several organ systems. Table 3 shows the results for all 38 non-overlapping types of cancer in which ≥5 instances of cancer were reported during follow-up.

**Table 3:** Observed and expected cancers of various type in Danish citizens with HS.

ICD codes	Cancer	Obs	Exp	SIR (95% CI)	*p*
**Any cancer**	**Any cancer**	**1193**	**881.47**	**1.4 (1.3–1.4)**	***
**C00–14**	**Oral cavity and pharynx**	**54**	**23.85**	**2.3 (1.7–2.9)**	***
C01–2	Tongue	8	4.35	1.8 (0.8–3.4)	
C03–6	Oral cavity	18	6.53	2.8 (1.7–4.2)	**
C09–10	Tonsils and oropharynx	16	8.17	2.0 (1.1–3.1)	*
C00, C07–8, C11–4	Other oral cavity and pharynx	16	5.97	2.7 (1.6–4.2)	**
**C15–26**	**Digestive organs and peritoneum**	**248**	**154.93**	**1.6 (1.4–1.8)**	***
C15	Esophagus	16	9.70	1.7 (1.0–2.6)	
C16	Stomach	17	12.82	1.3 (0.8–2.1)	
C18–9	Colon Including rectosigmoid	82	62.42	1.3 (1.0–1.6)	*
C20	Rectum excluding anus	52	33.37	1.6 (1.2–2.0)	*
C21	Anal canal	12	4.62	2.6 (1.4–4.4)	*
C22	Liver	20	7.28	2.7 (1.7–4.1)	**
C23–4	Gallbladder and biliary tract	11	4.46	2.5 (1.3–4.2)	*
C25	Pancreas	36	20.23	1.8 (1.3–2.4)	*
C17 C26	Other and poorly localized cancers of the digestive organs and peritoneum	7	3.51	2.0 (0.9–3.9)	
**C30–9, C450**	**Respiratory system**	**270**	**113.76**	**2.4 (2.1–2.7)**	***
C30–1	Nasal cavities and sinuses	6	1.76	3.4 (1.4–6.9)	*
C32	Larynx	9	6.14	1.5 (0.7–2.6)	
C33–4	Lung, bronchus and trachea	252	104.10	2.4 (2.1–2.7)	***
C37, C380–4 C388, C450, C39	Other respiratory system	6	2.78	2.2 (0.9–4.4)	
**C43–4**	**Skin**	**90**	**97.82**	**0.9 (0.7–1.1)**	
C43	Melanoma	55	68.99	0.8 (0.6–1.0)	
C44	Non-melanoma skin cancer	36	30.11	1.2 (0.8–1.6)	
**C451–9, C46–9, B210**	**Mesothelium of soft tissue**	**8**	**7.01**	**1.1 (0.5–2.1)**	
**C50**	**Breast**	**209**	**219.18**	**1.0 (0.8–1.1)**	
**C51–8**	**Female genital organs**	**73**	**83.09**	**0.9 (0.7–1.1)**	
C51	Vulva	6	3.53	1.7 (0.7–3.4)	
C53	Cervix uteri	22	26.08	0.8 (0.5–1.2)	
C54–5	Cervix	25	29.02	0.9 (0.6–1.2)	
C52, C56, C570–4, C58, C577–9	Other and poorly localized cancers of the female genital organs	21	25.57	0.8 (0.5–1.2)	
**C60–3**	**Male genital organs**	**59**	**62.23**	**0.9 (0.7–1.2)**	
**C64–8, D090–1** **D301–9, D411–9**	**Urinary tract**	**88**	**57.63**	**1.5 (1.2–1.9)**	**
C67, D090, D303, D414	Bladder	57	36.48	1.6 (1.2–2.0)	*
C64–6, C68, D301–2, D091, D304–9, D411–3, D417–9	Other and poorly localised cancers of the urinary tract	31	23.80	1.3 (0.9–1.8)	
**C69–72, C751–3, D32–3, D352–4, D42–3, D443–5**	**Eye, brain and other CNS**	**64**	**51.93**	**1.2 (1.0–1.6)**	
C70, D32, D42	Meninges	21	15.87	1.3 (0.8–2.0)	
C71, C751–3, D330–2, D352–4, D430–2, D443–5	Brain	36	25.99	1.4 (1.0–1.9)	
C69, C72, D333–9, D433–9	Other CNS	8	11.18	0.7 (0.3–1.3)	
**C73–4, C750, C754–9**	**Thyroid and other endocrine glands**	**10**	**12.66**	**0.8 (0.4–1.4)**	
**C81–5, 90**	**Lymphatic tissue**	**60**	**41.02**	**1.5 (1.1–1.9)**	*
C81	Hodgkin lymphoma	14	4.46	3.1 (1.8–5.1)	**
C82–5, C90	Non-Hodgkin lymphoma	46	36.57	1.3 (0.9–1.7)	
**C91–6, D459, D471, D473, D475**	**Blood forming tissue**	**46**	**33.54**	**1.4 (1.0–1.8)**	*
C91	Lymphatic leukemia	7	10.51	0.7 (0.3–1.3)	
C92	Myeloid leukemia	9	7.86	1.1 (0.6–2.1)	
D46	Myelodysplastic syndrome	5	3.89	1.3 (0.5–2.8)	
D471	Chronic myeloproliferative disease	8	2.77	2.9 (1.3–5.4)	*
D473	Essential thrombocythopenia	7	3.54	2.0 (0.9–3.8)	
C93–6, D474–5, D459	Other blood forming tissue	11	5.78	1.9 (1.0–3.3)	
**C76–80**	**Other and poorly localised cancers**	**50**	**24.55**	**2.0 (1.5–2.7)**	**
C77–9	Metastases and cancers of poorly specified tissue	40	18.67	2.1 (1.5–2.9)	**
C76, C80	Malignant neoplasms of other, ill-defined or unspecified sites	10	5.93	1.7 (0.8–3.0)	
**Basocellular skin cancer (not in C63)**	**Basocellular skin cancer - not included in any cancer**	**300**	**289.74**	**1.0 (0.9–1.2)**	

Follow-up starts 1 year after HS diagnosis. Cancer forms with obs < 5 are aggregated or not presented. Site-specific numbers may not add up to numbers in aggregated groups because individuals could only contribute once to the latter.

SIR is calculated as obs/exp with 95% confidence limits.

The asterisks signifies order of statistical significance: *p < 0.05, **p < 0.05/38 (thus accounting for the correction for multiple testing of the 38 presented non-overlapping cancer outcomes), and *** *p* < 10^-6^.

ICD: International Code of Diseases; CNS: central nervous system; HS: hidradenitis suppurativa; obs: observed number of incident cancers, exp: expected number of incident cancers; SIR: standardised incidence ratios.

### Oral cavity and pharynx

The risk of the combined group of cancers of the lip, oral cavity and pharynx was 130% increased (SIR = 2.3, 95% CI: 1.7; 2.9, *p* < 1 × 10^-6^), reflecting statistically significantly increased risks for cancers across most anatomical subsites (see Table 3 for all subgroup analyses).

### Digestive organs and peritoneum

Risk of cancers of the digestive organs and peritoneum was 60% increased (SIR = 1.6, 95% CI: 1.4; 1.8, *p* < 1 × 10^-6^), reflecting statistically significantly increased risks for all sites except esophagus, stomach and other digestive organs.

### Respiratory system

Risk of cancers of the respiratory system was 140% increased (SIR = 2.4, 95% CI: 2.1; 2.7, *p* < 1 × 10^-6^), reflecting statistically significantly increased risks of the nasal cavity and sinuses and for cancers of the lung, bronchus and trachea.

### Urinary tract

Risk of cancers of the urinary tract was increased by 50% (SIR = 1.5, 95% CI: 1.2; 1.9, *p* < 0.0005), which essentially reflected an increased risk of bladder cancer.

### Lymphatic tissue

Risk of cancers of the lymphatic tissue was increased by 50% (SIR = 1.5, 95% CI: 1.1; 1.9, *p* < 0.01), which reflect an increased risk of Hodgkin lymphoma.

### Other cancer types

Statistically significantly increased risks were observed for the group of metastases and cancers of poorly specified tissues. The risk of vulva cancer more than 1 year after HS diagnosis was not statistically significantly increased (SIR = 1.7, 95% CI: 0.7; 3.4).

### Further analysis based on HS severity

In our analysis of the effect of HS severity we found statistically significantly increased HR for all cancers (HR = 1.4, 95% CI: 1.2; 1.7, *p* < 0.001) (Table 4), cancers of the respiratory system (HR = 1.9, 95% CI: 1.3; 2.7, *p* < 0.002) and cancers of the urinary tract (HR = 2.1, 95% CI: 1.1; 4.0, *p* < 0.02) when comparing those with ≥5 HS-related visits and those with <5.

**Table 4:** Hazard ratios based on SIRs of various types of cancer measured in Danish citizens with HS when comparing ≥5 vs. <5 HS health-related contacts.

ICD codes	Cancer	HR (95% CI)	*p*
**Any cancer**	**Any cancer**	**1.4 (1.2–1.7)**	**
**C00–14**	**Oral cavity and pharynx**	**1.3 (0.5–3.2)**	
**C15–26**	**Digestive organs and peritoneum**	**1.1 (0.7–1.8)**	
**C30–9, C450**	**Respiratory system**	**1.9 (1.3–2.7)**	**
**C64–8, D090–1** **D301–9, D411–9**	**Urinary tract**	**2.1 (1.1–4.0)**	*
C67, D090, D303, D414	Bladder	2.2 (1.0–5.0)	*
**C91–6, D459, D471, D473, D475**	**Blood forming tissue**	**1.7 (0.7–4.2)**	

Follow-up starts 1 year after HS diagnosis.

HS: hidradenitis suppurativa; ICD: International Code of Diseases; HR: hazard ratios.

The asterisks signifies order of statistical significance: * *p* < 0.05, **p < 0.05/6 (thus accounting for the correction for multiple testing of the 6 investigated cancer outcomes) and ***p < 10^-6^.

## Discussion

In this nationwide register-based study on HS we observed a total 1,193 cases of cancer during 198,336 person-years of observation. This corresponded to a 40% increased risk of cancer among patients with HS compared with the general population. By far the largest of its kind in terms of follow-up time and number of cancers observed, our study both corroborates and expands on the earlier register studies of HS-related cancer risk (summarized in eTable 1). Thus, stratified analyses showed that the increased overall cancer risk reflected increased risks of a wide variety of different cancers.

For some of these malignancies, for example oral cavity and pharyngeal cancer, colorectal and liver cancer, and Hodgkin lymphomas, increased risks have previously been reported [15, 16, 22]. However, our analyses further suggest that persons with HS are also at an increased risk of cancers of the nasal cavity and sinuses, lung, pharynx and tonsils, anal canal, gallbladder, pancreas, urinary bladder, and other and ill-defined tissues. Of additional interest is the fact that patients with at least five HS-associated visits have a further increased risk of overall cancer, and of respiratory and urinary tract cancer in particular.

Controversially, in contrast to previous studies [15, 16], we found that the risks of non-melanoma skin cancer (NMSC) and prostate cancer matched those seen in general population, and while occurrences of brain cancers and non-Hodgkin lymphomas, were elevated, risk estimates were not statistically significant after CFMT.

One important point while addressing cancers within the HS population is that the chronic local inflammation seen in HS-affected tissues may itself contribute to malignancy. HS often affects the anogenital region [14, 23–25], and interestingly, we did find an excess of anal and rectal cancers, albeit not statistically significant after CFMT. Infection with Human papilloma virus (HPV) could represent a possible connection between HS and both perianal and vulvar cancer [14]. However, as the increased risks of anal cancers were not found accompanied by correspondingly increased risks of vulvar or other genital cancers, it appears that HPV-related cancers are not unusually frequent in the HS population.

Surprisingly, unlike previous studies [14–16] we did not find NMSC risk to be elevated among patients diagnosed with HS. Unfortunately, we cannot rule out the possibility of underreporting of NMSC to CAR in general [26–28] or for HS patients specifically due to NMSC’s usually inconspicuous appearance relative to the high impact symptoms of HS. However, an alternate explanation to our findings is that HS patients expose themselves less to sunlight due to social stigmatization and dread associated with undressing [29], thereby reducing their risk of this malignancy.

Finally, we found HS to strongly associate with risk of myeloproliferative disease. However, in and by itself this observation does not illuminate the controversy and uncertainties regarding the connection between these diseases. One potential consideration is that the constitutional immune-dysfunction inherent in HS may partly explain the elevated risk of myeloproliferative disease and lymphoma.

However, while the immune dysfunction in HS appears to be of a general nature and despite HS’s frequent clustering with autoimmune diseases [3, 6, 30–34], the fact that multiple organ systems contribute to the increased risk of cancer seen among patients with HS suggests that environmental causes should be considered. At a group level, HS patients are known to be encumbered by a variety of social and environmental risk factors. They are >4 times as likely to be smokers [12], >3 times as likely to be obese [4] and >50% more likely to have an alcohol abuse [3, 13]. For patients with severe HS, these figures are even higher suggesting dynamic interaction [12, 35]. This is important as patients with many consultations showed an additional increase in risk of all cancer and of respiratory and urinary tract cancer in particular (Table 4).

Smoking, obesity and alcohol consumption are of particular interest as they are all important environmental cancer risk factors [36] as well as actionable – thus presenting potential targets for intervention. Unfortunately, individual level information on these factors are not accessible through the Danish registries, so accounting for their effect were not possible during data analysis – an issue discussed thoroughly next.

Ultimately, both the site-specific cancers potentially attributable to HS-induced chronic inflammation and the malignancies attributable to environmental factors are theoretically preventable by HS disease control and life-style intervention. Unfortunately, many patients with HS have poor experiences with the medical community. Their HS diagnosis is on average made only after consultations with 3–4 different doctors [37]: a process often spanning 7–10 years [38, 39]. People with HS may therefore lack confidence in the healthcare system and be reluctant to seek and follow advice and/or treatment from healthcare providers [29, 40], a fact that is not surprising when considering the low HS disease awareness among physicians [41]. Indeed, despite a higher use of both emergency and surgical health care services [42], people with HS retain their risk behaviors in the form of smoking and obesity [4, 12].

Consequently, novel strategies are needed for life-style interventions to be successful in this young high-risk patient population. Information regarding a manifest increase in cancer risk may spur some patients to accept help in life-style intervention. Likewise, it may prove a valuable incentive for physicians to learn to recognize this often-overlooked disease as the population as a whole, for whatever reason, carries an overall increased risk of cancer of 40%.

## Strengths and limitations

The study’s greatest strength is the sound methodological design with register-based access to information on all hospital-based HS and cancer diagnoses from a nation-wide cohort during 40-years of follow-up. Furthermore, we mitigated spurious correlations between HS and cancer due to diagnostic work-up and treatment for one disease leading to the diagnosis of the other by only including cancers diagnosed at least 1 year after HS diagnosis in our analyses. Finally, all our analyses inherently adjusted for the effects of age, sex, and period on cancer risk in the Danish population.

The most substantial limitations of all register-based studies is of course the level and type of data accessible through the individual registers. First and foremost, the data quality of registers can be judged by their completeness and validity. Completeness is universally hard to determine, as the sensitivity of the physician-based diagnoses going into the register, and by extension the missed cases, is per definition unknown. The sensitivity for the HS diagnosis within DNPR has never been investigated, but it is likely low as HS is underdiagnosed since the disease is unknown by a majority of doctors [41, 43, 44]. While this indicate a low completeness, it conversely infers a high validity of the diagnosis simply because those diagnosed are likely to have received the diagnosis from a trained dermatologist, as other physicians are not familiar with the existence of HS. So far this has held true for the validity of the two dermatological diagnoses that have been assessed in DNPR, as these achieved positive predictive values (PPV) in the range of 77%–80% already at the first time of ICD diagnosis [45, 46] – a much higher PPV than the 73% normally estimated within the field of medicine [19]. Based on the aforesaid argument it is a qualified assumption that the HS diagnosis would achieve an even higher PPV than other dermatologic diagnoses upon first time registration.

In addition, of particular interest for this study, information on relevant environmental exposures was not available through the Danish registers. While Information on such environmental exposures are rarely, if ever, available through national-level health registers, it restricted our ability to elucidate how large a fraction of the cancer risk is attributable to carcinogenic exposures and to HS itself. While it is possible to attempt correcting for the effects of smoking and obesity by using surrogate measures such as ICD diagnoses of nicotine dependency or obesity the completeness and validity of these factors would be expected to be low. More importantly however, directionality and potential causality of high BMI and smoking upon the risk of HS still remain to be settled. Both smoking and obesity could therefore be potential effect-mediators of HS upon cancer risk, and therefore adjusting for their effect would be incorrect, as they would represent effect-mediators and not confounders [47, 48].

Lastly, the prevalence disparity between diagnosis-based register records and self-reported symptoms [7–9] suggests that those with more severe disease are the ones traditionally analyzed in the literature [49]. To inspect the effect of severity upon cancer risk we constructed a dichotomized variable for HS severity and through that showed that those patients with the highest number of HS-related contacts had a further increased risk of all cancer, particularly of respiratory and urinary tract cancer. It should however be remembered that this subgroup is likely to have a higher exposure to carcinogenic risk factor at group level [3, 4, 12, 13, 35] our findings may therefore not be equally applicable for all people with HS.

## Interpretation

We found people with HS to be at a markedly increased risk of cancer. While distribution of excess risk between exposures under the patient’s own control and those attributable to the disease itself remain undetermined, our findings emphasize the need for health intervention actively targeting this patient population of primarily young adults. Increased recognition and diagnostic ability among physicians, will be the first step toward better identifying this patient population at a high risk of developing cancer, while aid with life-style interventions constitutes a likely second step toward reducing this patient population’s increased risk of cancer.

## Data Availability

Authors K Rostgaard and H Hjalgrim had full access to all the data in the study and takes responsibility for the integrity of the data and the accuracy of the data analysis.

## Data sharing statement

Data from Danish registries are protected by the Danish Act on Processing of Personal Data and can only be accessed following application. Therefore, data sharing for this study is not possible.

## Additional information

The Department of Dermatology, Zealand University Hospital, Roskilde, Denmark, is a part of the European Reference Network for rare, complex, and undiagnosed skin diseases.

This study is supported by the BRIDGE – Translational Excellence Programme (bridge.ku.dk) at the Faculty of Health and Medical Sciences, University of Copenhagen, funded by the Novo Nordisk Foundation. Grant agreement no. NNF20SA0064340.
